# Beyond “study skills”: a curriculum-embedded framework for metacognitive development in a college chemistry course

**DOI:** 10.1186/s40594-022-00376-6

**Published:** 2022-09-24

**Authors:** Sonja Gamby, Christopher F. Bauer

**Affiliations:** 1 Natural Sciences, North Shore Community College, Danvers, MA 01923 USA; 2 Department of Chemistry, University of New Hampshire, Durham, NH 03824 USA

**Keywords:** Metacognition, Chemistry, Curriculum, Community college, Study skills, Self-regulated learning

## Abstract

**Background:**

There is a critical need for evidence-based metacognition instruction models with an ease of implementation. Three issues involved in advancing the implementation and assessment of metacognitive interventions are: (i) the lack of an operational framework for the development of metacognition; (ii) metacognition instruction models that lack a focus on explicitly engaging students’ self-perceptions; (iii) a lack of metacognitive interventions that are easy to implement and require minimal training. This study describes the development and implementation of a 10-week discussion-based module to promote metacognitive development as part of a general chemistry course at a community college. This curricular metacognition instruction model involved the explicit engagement of self-efficacy beliefs in addition to introducing metacognitive awareness and regulation through individual and group reflection. This approach involves a systematic framework which allowed students to confront their beliefs about their abilities, learn various task strategies, and practice these strategies along with their peers. This case study was designed to address the following: can explicit cognitive and metacognitive instruction and discussion serve as a catalyst for students to (1) build and adapt metacognitive knowledge about cognition, and (2) incorporate effective study strategies?.

**Results:**

Students’ individual and collaborative reflections were analyzed using a thematic analysis. Written journal responses indicate that the module facilitated a shared discourse about cognition where metacognitive awareness was observed shifting from a tacit to explicit awareness. In addition, the framework facilitated the formation of support networks (cognitive and emotional) where students were observed exchanging cognitive strategies and encouraging one another to persevere through challenges.

**Conclusions:**

Our findings suggest that the metacognitive instruction model described here can serve as a mechanism to encourage student reflection on their beliefs and behaviors. Instructors looking to include metacognition instruction could use the framework presented as a template. The discussion-based module is embedded in the curriculum, delivered through the course management system, and has a low barrier to implementation.

**Supplementary Information:**

The online version contains supplementary material available at 10.1186/s40594-022-00376-6.

## Introduction

“Before doing the reading or having any information about the brain, my mindset was very clear on the fact that there are two options out there: naturally smart or NOT.” —Student Reflection

There is widespread agreement that greater metacognitive skillfulness of students is beneficial for their learning outcomes (Askell-Williams et al., 2011; Veenman et al., 2006; Wang et al., 1990) and that an explicit focus on metacognition should be integrated into curricula (Ambrose et al., 2010; Benassi et al., 2014; Bransford et al., 2000; Schraw et al., 2006). In higher education, chemistry instructors have mixed opinions regarding when and how to integrate metacognition into curricula. Studies exploring the metacognitive skillfulness of university students reveal that many students rely on low-order study skills, such as rote memory and cramming (Chan & Bauer, 2016; Karpicke et al., 2009; Muteti et al., 2021). While there is limited information regarding the extent to which chemistry instruction is designed to develop students’ metacognitive abilities, a recent study involving instructors in Colorado (Heidbrink & Weinrich, 2021) supports the notion that chemistry instruction is not designed to develop metacognitive abilities. In the study, 17 instructors from six different institutions were interviewed to assess the extent to which they implicitly or explicitly incorporated a focus on metacognition. Nine of the 17 instructors interviewed believed they had some responsibility for developing their students’ metacognition, while only one instructor included an explicit focus on metacognitive development in their course. As metacognitive skills, including knowledge and regulation of cognition, develop through adolescence (Pressley & Schneider, 1997) and perhaps beyond (Verhaeghen et al., 1992), individual instructors may question the necessity and utility of including metacognition instruction in their courses for adult learners. However, as cited above evidence suggests that students are not acquiring this important development. Given the value that metacognitive skill provides to students, perhaps it is preferable that this important aspect of cognitive development is not left to chance. This article presents a framework for improving the metacognitive awareness and skillfulness of community college students enrolled in general chemistry. This framework was used to create a discussion-based module which integrated direct instruction with personal and collaborative reflection and supported students over the duration of the course.

### Metacognition in science education

Many researchers have investigated ways to bring the knowledge of learning and metacognition into science courses. This has been accomplished through either reflective practices or direct instruction. Reflective practices encompass approaches which are meant to promote a student’s reflection on their study habits and include activities, such as exam wrappers and participation in collaborative inquiry. Exam wrappers are surveys given to students after exams which require them to evaluate their performance and describe how they might change their study habits in the future. Lovett (2013) found that exam wrappers could improve course outcomes, but only when used in multiple courses. On the other hand, Gezer-Templeton et al. (2017) found a moderate relationship between grades and exam wrapper use after a single-use in an introductory nutrition course. Metzger and coworkers (2018) describe the development of a Student Metacognition Affect and Study Habits (SMASH) inventory which was used along with exam wrappers to encourage students to monitor their own learning in an undergraduate biology course. While the study was mainly descriptive, students’ course grades were correlated with how difficult they perceived the first exam to be. Others (Carpenter et al., 2020; Soicher & Gurung, 2016) have used exam wrappers with varying success.

Direct instruction has also been used as a mechanism to introduce metacognitive skillfulness. As the name implies, direct instruction involves explicitly teaching students metacognitive skills, such as effective study strategies, how to monitor their progress, and how to self-evaluate. Direct instruction can take place one time or several times throughout the semester. Cook et al. (2013) utilized a 50-min lecture on learning strategies to introduce metacognition and several study strategies. Students participating in the lecture earned an average course grade that was one letter higher (B versus C) than the quasi-control group. McGuire and coworkers (Zhao et al., 2014) repeated this approach by including a similar study skills lecture during class time. Students reported using higher level learning strategies and showed higher levels on the Chemistry Self-Concept Inventory (Bauer, 2005).

Other researchers have used direct instruction to focus specifically on certain metacognitive strategies. Casselman and Atwood (2017) focused on using instruction to improve general chemistry students’ abilities to predict their performance. Weekly quizzes and practice tests were administered to both an experimental and control group with students in both sections receiving detailed feedback on their performance and suggestions for how to improve. In the experimental section students were also asked to predict their performance and make a detailed study plan based on feedback regarding their performance and prediction accuracy. By the end of the course, the experimental group scored 4% higher than the control group on the final exam. In a recent study involving 259 undergraduate chemistry students, Muteti et al. (2021) reported that a significant percentage of students use ineffective study skills with 37.3% reporting that they use skills, such as reading and rereading, memorization, and cramming. After explicit metacognitive instruction delivered at various points throughout the semester, students reported using higher order skills more often. The authors concluded that it is imperative that students receive this type of instruction, and that continued focus should be placed on what this instruction looks like, writing: “[T]he scarce studies on metacognition instruction and models reveal the critical need for more tested metacognition instruction models to advance this research area.” (p.134). Inspired by this approach, Graham and coworkers (Graham et al., 2019) utilized chemistry tutors to deliver direct instruction to students in general chemistry. Students attended additional evening study sessions which introduced stategies such as interleaving, spaced practice, and dual coding while delivering chemistry lessons. This approach led to increases in self-efficacy as measured by the College Chemistry Self-Efficacy Scale (Uzuntiryaki & Aydin, 2009).  Improvement in self-efficacy were correlated with improved course outcomes and were more pronounced for women.

Of the approaches for integrating metacognition into chemistry courses presented in the literature, most have been directed at four-year university students. For example, in a recent review of metacognition in chemistry education Lavi and coworkers (2019) identified nine peer-reviewed articles involving metacognition and chemistry students in higher education between 2000 and 2018. None of these studies were conducted in a community college setting. In addition, these studies have generally involved the introduction of these skills via a short-term intervention. While there are instances where short-term interventions seem to be beneficial, the decision of students to opt-in suggests that their motivation positions them to take advantage of the opportunity. Furthermore, there is substantial evidence in other instances (e.g., professional development (Clary et al., 2018)) that short interventions may not be so effective for changing behaviors and beliefs. This study is novel in that it involves a longer-term, metacognition instruction module implemented in the community college setting.

### Acquisition of metacognitive skillfulness

**Fig. 1:**
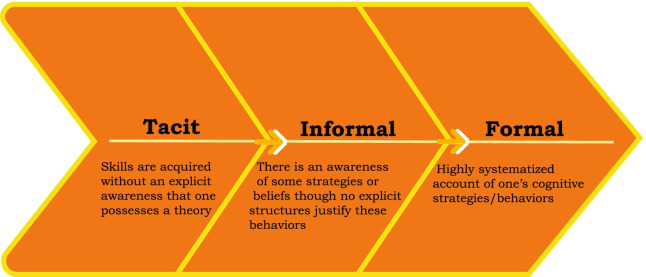
Schraw and Moshman model. According to Schraw and Moshman, metacognitive skillfulness is on a continuum. The three levels identified are separated by one’s level of awareness and control

When aiming to promote metacognition, it is necessary to consider how individuals consolidate metacognitive knowledge into personal metacognitive theories. Schraw and Moshman (1995) argue that cultural learning, individual construction, and peer interaction each play important roles in the emergence of metacognitive theories. Schraw and Moshman describe “cultural learning” as what has been described here as direct instruction. According to Schraw and Moshman, “cultural learning” describes how metacognitive theories can be internalized via formal instruction or informal experiences. The second component “personal construction” is the result of spontaneous, self-directed analysis of one’s own cognition. Lastly, “peer interaction” refers to the process of social construction as metacognitive conceptions are clarified among individuals in a peer group rather than being imposed by an expert (as in direct instruction). Tsai (2001) also describes this type of interaction among peers arguing that metacognitive improvement could be accomplished by “creating learning environments where students are allowed to explain and defend their thinking, opinions and decisions” (p. 972). While cultural learning, personal construction and peer interaction are essential elements of Schraw and Moshman’s theory of metacognitive development, they also argue that this development is dependent on an increasing recognition of one’s cognitive processes. According to this view, students possess theories which allow them to predict, control and explain their cognition or the cognition of others. Tacit theories are unconscious, unstated beliefs and behaviors that students use without recognition. Informal theories are intentional, personal, and fragmented. In this case, students may be aware of and consciously employ strategies that they believe to be effective, but their awareness arises from ad hoc personal experience and unguided skill development. Formal theories are expert-level: this includes awareness of an array of thinking strategies and monitoring and regulation in application. Thus, metacognitive ability is on a continuum, whereas learners progress from utilizing tacit theories which eventually develop into formal theories (Fig. 1).

The acquisition of metacognitive skillfulness is also deeply linked to self-efficacy, or one’s beliefs about their ability to succeed at a task. It has been shown that one’s beliefs about themselves as a thinker can impact their motivation (Diener & Dweck, 1978; Dweck & Leggett, 1988) and strategy use (Kizilgunes et al., 2009), whereas students with higher self-efficacy use deeper strategies for learning. Thus, it is critical when engaging with metacognitive instruction to explicitly address student beliefs about their ability to learn. The need to address beliefs within metacognitive interventions was described by Reeve and Brown (1985):

With few exceptions researchers interested in metacognitive development have ignored the influence of self-perceptions on performance. This seems an oversight since recent research ... suggests that individuals’ assessment of their own abilities affects performance on cognitive tasks (p.351).

Though Reeve and Brown (1985) asserted this over three decades ago, an emphasis on student beliefs is apparently still lacking in the literature. In fact, this need was echoed more recently by Lovett (2008). Despite these suggestions, the literature contains many examples of metacognitive interventions that lack explicit focus on students’ self-perceptions about their ability to learn. For example, none of the interventions described previously included an explicit emphasis.

### Research goals

The goal of this study was to design and evaluate a metacognition module that serves to instruct students directly regarding metacognitive knowledge and to engage students’ beliefs about themselves as learners while encouraging an environment which supports individual and collaborative reflection. The efficacy of the proposed framework is evaluated by the extent to which it facilitates the acquisition of metacognitive knowledge and skills.

## Methods

### Framework for promoting metacognition

Flavell (1979) is credited with introducing the term metacognition, which is regarded generally as thinking about one’s thinking. Flavell asserted that there are four components of metacognition—skills, actions, knowledge (beliefs), and experiences—and that these are necessarily interrelated. For example, as one regulates a certain cognitive skill (metacognitive actions) they are necessarily drawing on previous metacognitive knowledge and experience. Metacognition is described in various ways by others (Nelson, 2000; Reeve & Brown, 1985; Schraw & Moshman, 1995), but the general sense is that metacognition is a process by which an individual monitors and controls their own learning. This can be broadly described as requiring knowledge of metacognition and metacognitive skillfulness which includes assessing a task, evaluating (both the task and one’s strengths and weaknesses), planning, applying (and monitoring) strategies, and reflection. The process is iterative and thus, the methods a student uses are expected to change over time or when performing different tasks. Accordingly, metacognitive knowledge of self (epistemological beliefs), task, and strategy is essential to improved metacognitive regulation.

Given the nature of metacognition, it reasons that one could come up with an exhaustive list of metacognitive characteristics to investigate. This is in fact what has occurred. Veenman et al. (2012) describe this problem, writing:

Under the umbrella of this inclusive definition a proliferation of metacognitive terms has unfolded through the years. Metacognitive beliefs, metacognitive awareness, metacognitive experiences, metacognitive knowledge, feeling of knowing, judgment of learning, theory of mind, metamemory, metacognitive skills, executive skills, higher-order skills, metacomponents, comprehension monitoring, learning strategies, heuristic strategies, and self-regulation are several of the terms we commonly associate with metacognition. While these terms emanated from and helped to focus research, the domain of metacognition is one that lacks coherence (p.4).

As described above there is a need for a more operational definition of metacognition which identifies essential components. Thus, the challenge is to identify a set of characteristics that is comprehensive yet also concise. The social cognitive perspective of self-regulated learning presented by Zimmerman is a useful one through which we can view metacognition.

**Fig. 2:**
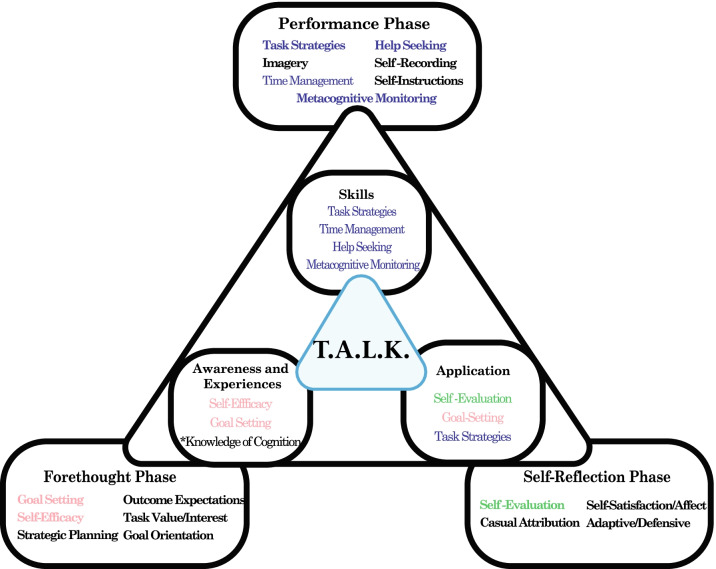
Metacognitive framework. The outer triangle represents the iterative process as described by Zimmerman. The framework described in this study utilizes a selected subset (inner triangle) of these attributes consistent with Flavell’s four components of metacognition. Attributes in pink, blue and green were selected from the forethought, performance, and self-reflection phase, respectively. Two of Flavell’s constructs “metacognitive experiences” and “metacognitive knowledge” are taken together as “Awareness and Experiences”

Zimmerman’s (1989) definition of self-regulation as, “self-generated thoughts, feelings, and actions that are planned and cyclically adapted to the attainment of personal goals” is very similar to Flavell’s definition of metacognition. In this perspective, “self-generated thoughts” are comparable to metacognitive knowledge, “feelings” would equate to Flavell’s experiences, and “actions that are planned and cyclically adapted” would correlate to Flavell’s metacognitive goals and actions. Zimmerman’s cyclical process (Zimmerman, 2000) is defined by three phases: forethought, performance, and self-reflection (Fig. 2, outer triangle). To establish a practical framework for metacognitive development, a subset of attributes in Zimmerman’s model was selected in this study to address each of Flavell’s components of metacognition (Fig. 2, inner triangle). Included in this framework is an additional attribute—knowledge of cognition. This attribute was added to promote an increasing recognition of cognitive strategies. Together, these nine attributes describe the basis of the metacognitive training implemented in the instructional framework described in this article. The following section will explain how these attributes are introduced to students in a sequential manner in three phases: Awareness and Experiences, Skills, and Application.

### The talking about learning is key (TALK) module

A module entitled—TALK—Talking About Learning is Key (Additional file 1), was created to systematically guide students through understanding their cognitive and metacognitive processes, and to encourage reflection on these processes. The module consists of three phases which aim to:

1. Encourage students to reflect on how they view themselves as learners and on learning as a process.
2. Introduce students to metacognitive strategies.
3. Provide an opportunity for students to practice strategies in a chemistry context.

A discussion-based module (Table 1) was created to allow for both personal and collaborative reflection. Phase one (weeks 1–3) of the module begins with topics involving self-efficacy beliefs and goal orientation before gradually introducing students to cognitive science and the concept of metacognition. Phase two (weeks 4–7) focuses on specific task strategies including reading strategies, note taking, concept maps and goal setting. During phase three (weeks 8–10) students are tasked with completing chemistry assignments while using previously introduced strategies. The module was implemented though the Learning Management System (Blackboard) utilizing the discussion board feature. Students were presented with video and/or written resources as direct instruction which introduced various cognitive concepts as the discussion topic. Students were asked to respond to prompts by reflecting on their awareness and/or use of concepts (or strategies) and asked to share their general impressions. They were also required to reply to at least one peer.

**Table 1:** Summary of the TALK module

Phase	Purpose	Metacognitive component attribute
**Phase I: *Awareness and Experiences*** Weeks 1–3	To engage students’ beliefs about themselves as learners and about effective study strategies To encourage students to reflect on experiences they have had while learning and introduce students to metacognition	Metacognitive Knowledge/experiences • Self-efficacy • Nature of intelligence • Goal orientation • Knowledge of cognition—introduction to cognitive science/metacognition
**Phase II:** ***Skills*** Weeks 4–7	To introduce selected metacognitive learning strategies To encourage students to reflect on learning strategies they have used in the past	Metacognitive Skills • Task strategies—includes reading strategies, concept maps, note taking, study cycle and directed paraphrasing • Time management • Help seeking • Metacognitive self-monitoring
**Phase III:** ***Application*** Weeks 8–10	To practice previously introduced strategies with chemistry content	Metacognitive actions • Goal setting—revising goals and adjusting schedule based on performance in course • Self-evaluation—practice with self-assessment methods while solving chemistry problems • Task strategies—practice constructing concept maps and using directed paraphrasing

The framework presented here incorporates each of Flavell’s components (right column) into three phases (left column)—Awareness and Experiences, Skills, and Application. Selected attributes identified by the authors are placed into each of these three phases

As an example, during week two students complete a discussion activity entitled, “*What is your model describing your ability to learn*?” Students read an article (*You can grow your intelligence*, MindsetWorks) describing how the brain grows and creates new neural connections as one learns new information. The article provides direct evidence describing how connections in the brain are multiplied, and references studies contrasting the brains of animals who lived alone and were deprived of toys with those of animals who lived with other animals and were presented with challenges and opportunities for play. The video resource was a review of Carol Dweck’s “Mindset” (Dweck, 2006) including research describing how fixed and growth mindsets lead to different goal orientations and ultimately different outcomes. Students were asked to reply to the following discussion board questions:

1. *Were you familiar with the idea of the brain growing in response to stimulation before reading the handout?*
2. *Describe any initial impressions you had about the reading. For example, did you feel encouraged or about the same as before? Do you have any questions about the topic?*

Discussion posts were treated as participation and worth 5% of the students’ overall course grade. As the authentic engagement from students in online discussions was a focus of this study, posts were graded for completeness and relevance. In the grading rubric students were reminded that they could “agree, disagree or anything in between so long as all replies are courteous and respectful.” When grading posts, the instructor provided general feedback to individual students, such as “Well said, [student name]”, “Good question!” or “Great post. Don’t forget to reply to a peer”. Instructor participation in the online forums was limited to private feedback to individuals to preserve the online space for student-centered interactions. A complete description of each week of the module along with a rubric used for grading is available (Additional files 1 and 2). The module was piloted in a general chemistry course during the fall 2018 semester. The main objectives of the pilot test were to assess student buy in and to calibrate time of administration. The data reported here are from the Spring of 2020.

### Participants

Participants (*N* = 18) were enrolled in a section of the first semester of a general chemistry sequence at a Community College in Massachusetts during the Spring 2020 semester. The College has an annual enrollment of just over 6000 students, with 64.4% of students attending part-time. Roughly 59% of students are between the age of 18 and 29, while just over 32% of students are over 25 years old. Students enrolled in general chemistry are by and large science majors pursuing biology, chemistry, and engineering biotechnology programs. General chemistry courses run as a single section with the same instructor teaching the lecture and laboratory components. Sections typically include 20 students. Due to the COVID-19 pandemic face-to-face instruction was moved online during week five of the module. Overall, ten participants provided consent and completed the course. The remaining students either withdrew (*N* = 3) or stopped attending (*N* = 4). IRB approval was obtained for study, and informed consent was obtained from students for their anonymized information to be published; all names used are pseudonyms.

### Research questions

The literature supports the role of metacognition in learning, but interventions have had mixed levels of success. There is a clear need to identify essential elements for practical implementations of metacognition instruction. As described above, a discussion-based module was developed to explicitly address each theoretically essential aspect of metacognition. The focus of this study involves the following research questions:

1. *To what extent can explicit cognitive and metacognitive instruction and discussion serve as a catalyst for students to build and adapt metacognitive knowledge about cognition?*
2. *To what extent can explicit cognitive and metacognitive instruction and discussion serve as a catalyst for students to adopt effective study strategies?*

### Data collection and analysis

Data sources included surveys, discussion posts and replies. An exit survey (Additional file 3) was used to capture students’ experiences and to determine if students reported changes in their study habits or strategy use. In discussion posts students commented on the metacognitive module and/or indicated their awareness of metacognitive applications or skills. Responses from peers were also considered to characterize how students interacted with each other.

Textual data were coded from two perspectives: theoretical considerations regarding metacognition, and emergent salient issues. For example, the deductive code “awareness” arose out of the theoretical importance of metacognitive knowledge while the inductive codes “intentionality” and “negative self-thoughts” arose from the discussion responses (Table 2). The first author independently established codes (Additional file 4) and conducted the initial coding using NVivo 12 (2020). Next, the corresponding author used the codebook to categorize student comments independently. This second coder was not involved with participants, with data collection, or with developing curriculum materials. Both authors then compared codes on a week-by-week basis, negotiating to a common meaning over subsequent weeks. Finally, a third coder (graduate student, not involved with the project) was recruited to evaluate inter-rater reliability with a sample (10%) of the data. After an initial coding, the proportional agreement was 60%. An analysis of the disagreements revealed that most of the disagreement originated from three related codes—intentionality, identifying a challenge and evaluating. Excluding these codes Cohen’s Kappa calculation suggested a moderate agreement (κ = 0.74 ± 0.17, 95% CI) with a proportional agreement of 80%. It was found that the differential interpretation of these three related codes was heavily dependent on context. Thus, it was decided to merge them into a more general code (appraising) which yielded a higher level of agreement. This resulted in a net moderate agreement (κ = 0.68 ± 0.13, 95% CI) with an overall proportional agreement of 76%.

**Table 2:** Some examples of inductive and deductive codes along with representative statements from students

	Representative student comments
Inductive codes	
*Negative self-thoughts* Describes items in which students make comments that diminish their intelligence or abilities, or, express being frustrated with their performance *I should try that* Describes items in which students respond to a strategy posed by a classmate and express a desire to incorporate said strategy *Supporting and encouraging* Describes items in which students offer assistance or encouragement to one another	“I was uplifted as I have struggled in the past with motivation and becoming overwhelmed, and the idea that “I can’t do it” can become a daily thought.” “It’s interesting that you talk to yourself out loud when doing homework. I wonder if it helps you catch mistakes because you have to think more deliberately about what you're doing? This is probably something I should try and see if it helps me.” “I think your mindset is the best one you could have right now, it is interesting that once we stop doing something, we lose that skill, although it makes sense. However, I think if you keep going and not give up, you will do whatever you put mind into. Good luck !”
Deductive codes	
*Awareness (* ±*)* Describes statements indicating the awareness of a cognitive concept or strategy	“I was not familiar with that concept. It is very interesting and makes perfect sense “[T]here were a ton [of strategies] that I was not familiar with.”
*Use (* ±*)* Describes statements indicating the use of a strategy	“I currently do not preview the material before lecture.”

The complete codebook can be found in Additional file 4

**Fig. 3:**
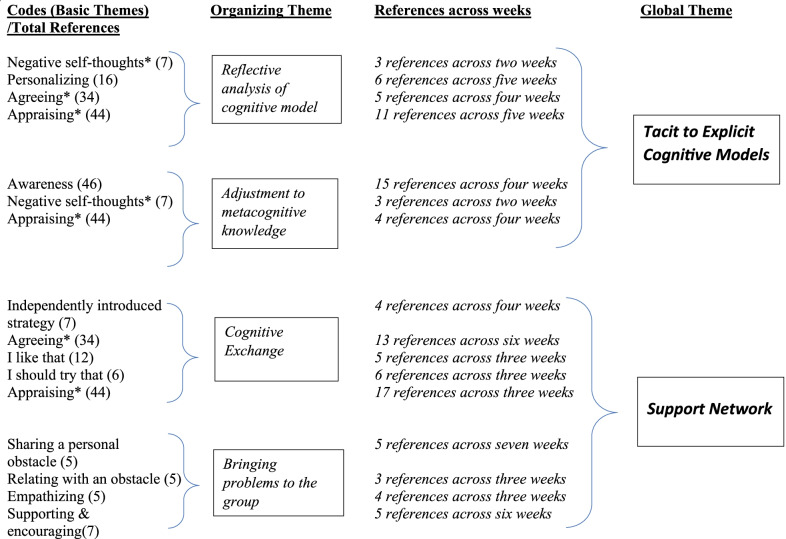
Thematic analysis via Attride-Stirling. Basic themes were used to group student statements more broadly into organizing themes and again into global themes. The first column on the left provides the total number of references for basic themes while the third column provides references of those basic themes within specific organizing themes. (*The basic themes awareness, agreeing and negative self-thoughts occurred across more than one organizing theme.)

Written artifacts were analyzed using a thematic analysis as described by Attride-Stirling (2001). Briefly, basic themes were derived from codes and formed the basis for organizing themes which were themselves further combined into more unifying global themes. The process evolved with each week of data in that text fragments containing basic codes were analyzed and grouped more broadly into organizing themes which persisted from week to week. Once organizing themes were identified, they were coalesced again into broader global themes (Fig. 3). A description of the global themes created from these networks follows. All student comments have been reproduced in their original form unless otherwise indicated.

## Results

### Research Question #1:

To what extent can explicit cognitive and metacognitive instruction and discussion serve as a catalyst for students to build and adapt metacognitive knowledge about cognition?

Throughout the 10-week module, students made comments indicating that there had been changes to their knowledge and beliefs regarding cognition. This is described by the global theme, *Tacit to Explicit Cognitive Models*.

### Global theme: tacit to explicit cognitive models

As the name implies this theme represents the apparent transition of students’ metacognitive knowledge from unconscious to explicitly understood and deliberated. This global theme represents the combination of two organizing themes, *Reflective Analysis*, and *Adjustment to Metacognitive Knowledge* (Fig. 3) which include basic themes*: negative self-thoughts, personalizing, agreeing, appraising,* and *awareness.* As an example of this global theme, consider Erin’s statement discussing a recommended study cycle that emphasized previewing and reviewing.

Erin: I currently do not make a point of doing a “preview” before lectures. If I have time to spare before class and I know there is a specific powerpoint or something like that that’s going to be used in the class, I will sometimes try to look at it beforehand. I definitely think it’s a good idea to consistently set aside 5–10 min before lecture to look over what the lesson will be about. I can see how it would help me get more out of the material. I think the biggest issue would be consistency. Once I establish a routine, I’m pretty set in it, but getting there is always a challenge for me. In the future I’ll try to make a point of “previewing” before class, especially once physical classes resume. I’ll probably try to attach “previewing” to another task in the meantime, such as making a coffee before schoolwork time. Attaching a task to another task is usually how I find the most success in building a habit.

Erin’s statement implies that they previously placed little value on previewing; it is something they do if they have “time to spare”. After completing the exercise, the student describes a more formalized awareness of the use of this strategy **{Basic theme: *awareness*}** and indicates the value (“I can see how it would help me get more out of the material”). They weigh the benefits of previewing against their perceived obstacles in establishing this routine **{Basic theme: *appraising*}** while planning on how to implement it in a sustainable way **{Basic theme: *personalizing*}.** Taken together the statements are an example of one of the organizing themes (*Reflective Analysis*). Additional examples of the organizing theme *Reflective Analysis* are discussed below.

### Organizing theme: reflective analysis

This theme describes the deliberation of cognitive strategies or concepts and includes the basic themes: *negative self-thoughts, personalizing, agreeing, appraising.*

### Basic theme: negative self-thoughts

At times students expressed self-deprecating comments while considering concepts and strategies.

Darnell*:* Self-evaluation is a great way to pinpoint personal goals and struggles, but I’ve never really done it that much. I think I tend to underestimate my ability to understand things and I get really frustrated trying to get through things and therefore hinders my ability to learn.

Billie (responding to Darnell): I can relate to the sentiment of hindering your own ability to learn with frustration. I don’t hold on to information well, and having to figure something out again in order to progress on later topics can leave me feeling upset with myself, which only makes the process take longer. The discouragement can have an impact on even my other classes which I am more confident in. Still, I hope that we manage to find appropriate study methods which help us to comprehend everything and, over time, we are both sure to stop needing to go back and brush up on the simpler elements.

In the comments above, students are expressing negative emotions, such as getting “really frustrated” and “feeling upset”, while reflecting about how these feelings can “hinder” their ability to learn or “have an impact on other classes”.

### Basic theme: personalizing

This basic theme describes statements which indicate that a student connects with material in a personal way as demonstrated by recalling a previous experience or discussing how a concept or strategy is relevant to or could be integrated into their life.

Sue: I chose to write about the third key point in the article about metacognitive learning. It was never obvious to me growing up, partially because I was confident in the subjects that came naturally to me and didn’t care enough about the ones I struggled in. Today however, I guess I’ve been using a number of strategies to monitor and better my understanding of things. I think any time we supplement a lecture or reading with outside resources or search for someone to expand or explain a topic until it clicks, that we are taking a metacognitive approach.

In this quote, Sue is recalling past academic experiences as they were “growing up” and is describing how they have become more metacognitively engaged. Sue is connecting personally with the concept as they explain how they previously “didn’t care enough” about courses which were more of a struggle.

Billie shares a personal anecdote while discussing strategies for memorizing facts.

Billie: Understanding the bigger picture, beyond what is necessary, makes the content go from overwhelming to feeling simple and straightforward- like there’s a logic behind it…I also like to link silly words to things and paraphrase to friends. Sometimes the actual meaning doesn’t stick as well as goofy conversations. I passed my bio final because of Sherlock, soap, and cheerios.

### Basic theme: agreeing

This theme describes how students reflected on strategies in response to a peer. In these instances, students went beyond simple agreement and elaborated on the strategies in question. Regarding metacognition, Colin responds to a peer writing, “[I] think you are so right about how people monitoring their own thinking can improve one's academic performance. Its like getting information and translating into your very own brain language, and if you understand it is way easier to apply the information learned into any situation.”

When discussing reading strategies, several students discussed “zoning out” or hitting “lulls” when completing reading assignments. Asked if they planned on adopting any strategies that were discussed, Michael states: “I think I could benefit from underlining and circling items in the text. This could allow me to remember certain aspects of the text.

*Morgan:* I agree that it can be pretty easy sometimes to be reading a chapter and realize once you’re 2/3 of the way through “what did I just read?”. That has always been one of my worst problems, but I feel like if you’re highlighting and circling important things as you go, like you said, than this would become less of an issue.

During week one and the discussion around grit, Keira recognizes that it is “hard to have the stamina to envision yourself completing something years in the future.” Replying to Keira, Colin writes:

I totally agree with you and the fact that it is hard to find the stamina to do something. We all want everything to come easy to us, but I also think in today’s society everyone tells us to do things that are so easy to say but so hard to do. Especially with the older generation, everything looks easy to them, when we are just trying to not drown in this society and its expectations. Also, grit could be the solution to all our problem but we have to think about how are we going to start doing something we have never done before. And how to teach our brains to change the way of thinking.

In the comments above, we can see that students are not only agreeing with the sentiments expressed by their peers, but also expanding on strategies and concepts that are introduced.

### Basic theme: appraising

This theme describes statements in which students are apparently weighing the potential benefits of adopting a strategy or concept. This happened in several ways and included statements in which students evaluated the benefits of using a strategy, discussed the intentional use of strategies, or recalled a past academic challenge while discussing a strategy. For example, when describing previewing as a strategy, Darnell and Colin appear to evaluate the benefits of previewing material before lecture.

Darnell: I do preview before lectures but it’s not a consistent thing that I do. It’s something that really does help me, so one could ask why don’t I do it more often. Knowing what will be covered makes it a lot easier to understand information as opposed to just hearing and seeing new information for the first time in class.

Colin: Until recently, I actually rarely previewed because I want the information to get into my head as the professor’s version, so I don’t confuse my version of understanding with the real version. However, especially with this semester, I’m more than considering previewing because I feel like I need another technique and this one sounds pretty good to me.

During week four students took a Metacognitive Reading Awareness Inventory (Mokhtari & Reichard, 2002) and discussed their awareness and use of strategies. Some students made statements indicating that they were evaluating the potential benefit of adopting certain strategies.

Sue: While there are quite a few strategies that I don’t think I will ever use when reading a textbook, the survey shed some light on a few that I will try to use. I think taking more side notes/summaries could be useful and while I do preview what the chapter will be about, I don’t do it with the intention of setting myself up to absorb the info…

The statements above involve the deliberation of cognitive strategies. Darnell pondered why they wouldn’t preview before class when it “really does help”, while Colin expressed that while they had reservations about previewing in the past that now they “need another technique”. Sue acknowledges that while they do preview, they are not doing so with intention.

At other times during the module students recalled an academic challenge when discussing strategies. For example, Sue reflects on one of the reading strategies saying, “I also like the idea of having a purpose when reading, too much of the time reading a textbook becomes monotonous and reading with intention may be able to clear that up.”

Billie (replying to Sue): I also found that reading with intention could be a strategy worth trying to employ. The idea of taking notes for what those intentions are would be even better, since it would force me to articulate exactly what I’m trying to learn rather than going in with vague, mushy concepts (which I’ll admit, I'm very prone to doing**)**

Tony (replying to Sue): I really like what you said in the last part. A textbook is such an overwhelming amount of information and having some kind of direction for what’s important and what isn’t as essential is really helpful. I think that textbook reading can be extremely monotonous and I always reach a point where I kind of hit a wall and can’t absorb or really comprehend anything that I'm reading anymore.

Here Billie describes approaching texts with a vague idea of what the material is about, while both Tony and Sue describe “zoning out” or “hitting a wall” when reading the textbook becomes “monotonous”. In these cases, students are suggesting that a certain strategy could help remedy a perceived problem.

In some instances, students expressed a desire to intentionally use a strategy or implied a value in intentionally being aware of a concept. An example of this is when Erin completes the reading strategies inventories and notes that they don’t use any of the strategies consistently.

Erin: While taking the quiz I noticed that there are a lot of strategies that I use from time to time while reading for schoolwork, but very rarely do I use any strategies consistently. I think that to be a better student and get more out of my time with the text/material, it would be helpful for me to deliberately commit to using a few strategies, and try to build it into more of a habit. I think I might write a few of my favorites that seem the most useful down on a post-it and stick it inside my textbook, or to my laptop or something at home. This way I’ll go into reading with an overview of what I need to do to get the most mileage from it.

In the statements above we can observe Erin’s behaviors becoming more formalized as they assess the ability of certain strategies to help them “get more” out of their time. Throughout the module students made statements appraising strategies presented to them. These instances involved students selectively discarding some strategies while planning on integrating others and are consistent with the organizational theme, *Reflective Analysis*.

### Organizing theme: adjustment to metacognitive knowledge

*Adjustment to Metacognitive Knowledge* describes students’ statements which imply an addition to or a shift in metacognitive knowledge. This theme includes the basic themes *awareness, negative self-thoughts, appraising.*

### Basic theme: awareness

Students frequently expressed the fact that they were being made aware of a concept or strategy for the first time.

Michael: Overall, I learned a lot after watching the video. I never thought about what traits make a successful person. It was surprising to learn that the ability to have grit was a positive attribute in every field Duckworth looked at.

Ana (replying to Michael): I agree with your post too and second all you have said. I too never knew that it took grit to succeed. I used to think success can be circumstantial and that it did indeed depend on intellect. Very interesting video this was.

In the discussion above Michael acknowledges having never thought about what makes a person successful while Ana had previously viewed success as circumstantial. In both cases the students have gained a new awareness regarding the effort required to accomplish their goals. This was also observed as students discussed mindsets and brain development.

Erin: That’s really interesting. I had never really heard about the idea of the brain creating new pathways in response to stimuli, so this was a really new thing for me.

Ana: I was not familiar with that concept. It is very interesting and makes perfect sense. The more you do anything, the better you can get at it. I like the example about the animals who played with toys verses those who only ate and slept… The idea makes sense to me, but I never thought about it this way until now.

When discussing the idea of metacognition, the role of preconceptions in learning, and the importance of deep factual understanding/chunking during week three, several students indicated that these ideas were not necessarily obvious to them. However, other students did recognize these principles after being formally introduced to them.

Tony: Of the three main points discussed in the text, I am interested in the megacognitive strategies. I know everyone has different learning styles and I know that when I'm doing homework, I like to talk to myself … After reading the article, the points discussed seem obvious and make sense, but before reading the article, I hadn’t given it much thought. I guess being cognizant of these things could even help to improve thinking and learning.

Tony’s statement represents the transition from implicit to explicit knowledge of a cognitive strategy, writing that after reading the article, “the points discussed seem obvious and make sense” though they acknowledge not having previously given it thought.

Erin (replying to Tony): I thought the same thing; before reading the article I hadn’t really thought about this, but after, it seemed like something worthy to be aware of. I can definitely see how this information could be helpful when going forward with studying/learning new things. It’s interesting that you talk to yourself out loud when doing homework. I wonder if it helps you catch mistakes because you have to think more deliberately about what you’re doing? This is probably something I should try and see if it helps me. I often find myself making dumb errors when I'm working because I tend to lose focus and let my mind wander. Could help me stay more on task **{also, basic theme: *appraising*}**.

According to statements above there were aspects about the cognitive process that some students have been aware of implicitly, though they lacked an explicit awareness. The discussion prompted students to reflect upon and make sense of previous experiences while also considering the future application of the concepts that were introduced. For example, regarding metacognition, Erin specifically addresses the recurring issue of losing focus and making “dumb errors” while acknowledging that a metacognitive approach could help them “think more deliberately” and stay on task. Collectively these statements describe either the addition and/or adjustment of metacognitive knowledge, and illustrate the organizational theme, *Adjustment to Metacognitive Knowledge.*

### Basic themes: negative self-thoughts and appraising

As described previously, *appraising* includes statements in which students are discussing the potential benefits of adopting a strategy or concept. Within this organizing theme, statements coded under *appraising* include statements in which students are apparently evaluating their previously held beliefs. This was also accompanied by *negative self-thoughts* in three instances. For example, Colin is observed evaluating their beliefs regarding mindsets and brain development, writing:

Before doing the reading or having any information about the brain, my mindset was very clear on the fact that there are two options out there: naturally smart or NOT. But this way of thinking is so wrong and I don’t think the whole world understands it yet. A lot of people still, including myself, always had the mentality that to be successful, academically or not, you need a smart brain and there is no option of “growing one”. Now, this idea is changing slowly but surely, I realize that your brain is actually a muscle, you just need the right work

This sentiment is echoed by Jan who expressed changing the way they “accept challenges”.

Jan: I was aware of the brain always growing in response of learning, although I did not have much knowledge concerning the two different mindsets people have. The reading plus the video helped me expand the way I learn and accept challenges... I felt more encouraged to improve my learning styles and the way I view challenges. Instead of not accepting a challenge solely based on being afraid of failure or the risk of failing, I should feel more positive with whatever the outcome is as long as I tried my best.

Darnell also implies they are evaluating their beliefs when discussing grit and factors that play a role in success.

I loved this Ted talk as it put a perspective on how I learn. When I don’t understand a concept, I usually let that get in the way of my future success in that subject. I feel like I have to be good at that subject from the beginning and if I don’t, then I’ve failed. This video has given me a renewed sense of hope in doing well in chemistry.

Here Darnell has identified a negative attitude explaining that not understanding a concept gets in the way of their “future success” while Jan implies that they have previously been “afraid of failing”. Colin summarizes these sentiments saying, “[b]ut this way of thinking is so wrong”. Both students indicate that they now feel “encouraged” with a “renewed sense of hope”. Taken together the statements presented under *Adjustment to Metacognitive* Knowledge illustrate how students have both incorporated new strategies and adjusted non-productive beliefs.

### Research Question # 2:

Can explicit cognitive and metacognitive instruction and discussion serve as a catalyst for students to incorporate effective study strategies?

During the 10 weeks of discussions in the forum students interacted with each other sometimes bouncing ideas off one other. During this time, students made statements that indicated they were not only considering adopting strategies presented but also those suggested by their peers.

### Global theme: support network

Throughout the module students provided personal reflections and interacted with each other as they replied to each other’s posts. *Support Network* (Fig. 3) is the global theme arising out of the content of these interactions. These interactions occurred in various ways that could be broadly generalized into two organizing themes: *Cognitive Exchange* and *Bringing Problems to the Group* and included basic themes which include: *independently introduced strategy, agreeing*, *I like that, I should try that, appraising, sharing a personal obstacle, relating with an obstacle, empathizing,* and *supporting & encouraging.*

### Organizing theme: cognitive exchange

Students’ peer-to-peer interactions were not restricted to the strategies and concepts which were introduced. At times students posed solutions to problems shared in the forum and shared strategies that they have found to be effective. The organizing theme *Cognitive Exchange* describes these types of exchanges and includes basic themes: *independently introduced strategy, agreeing, I like that, I should try that,* and *appraising.*

### Basic themes: independently introduced strategy and agreeing

At times students elaborated on strategies which were introduced and offered some practices of their own. On some of these occasions, other students resonated with the techniques shared by their peers. When responding to whether they self-evaluate, Tony goes into detail to explain their process of self-evaluation.

Tony: The “assess” portion of the cycle is something I definitely employ while studying. Whenever I am reading or doing practice problems, I try to think about my thought process (so that I can have a thought system in place) and the bigger concepts surrounding the problem/topic. I also try to think of ways the problem could have been changed to make it more difficult so that I don’t get tripped up on future problems (**Basic theme: *Independently introduced strategy***).

Morgan: I agree that it can be hard when just looking over notes to find your focus (**Basic theme: *agreeing***)… When that happens to me I tend to get discouraged, but its just about staying busy with it instead of just reading it or looking at it. Maybe do a different version of the same problem. I totally agree that writing down or thinking of different numbers and examples of previous problems can help understand all of the individual aspects of it.

### Basic themes: I like that and I should try that

As the names suggest, *I like that* and *I should try that* describe instances in which students express liking or wanting to try a technique that one of their peers introduces.

Michael: I have evaluated the methods I use to study. I have adjusted these methods throughout this course. For example, I now spend more time on practice problems. I found doing practice problems was an active way of studying vs passively reading the textbook.

Billie (replying to Michael): I like your method of focusing more on the practice problems than the passive reading. I find too often that I lose focus and can’t extrapolate what I need to from the written text without sitting down with a tutor or waiting for lecture to explain it to me in clear terms. If I were to read the practice problems and try to solve them as I went, I think I could have an easier time with my weekly objectives **{Also, basic theme: *appraising*}**.

In this example, Michael discusses how they have evaluated their method for studying noting that they have prioritized actively studying (by doing practice problems). Notably, this is before self-evaluation was discussed. Billie responds by acknowledging that they tend to lose focus while reading the text and offering that focusing more on practice problems could be a solution. While Billie does not indicate whether they will adjust their study habits, they are apparently evaluating the benefit of doing so.

**Fig. 4:**
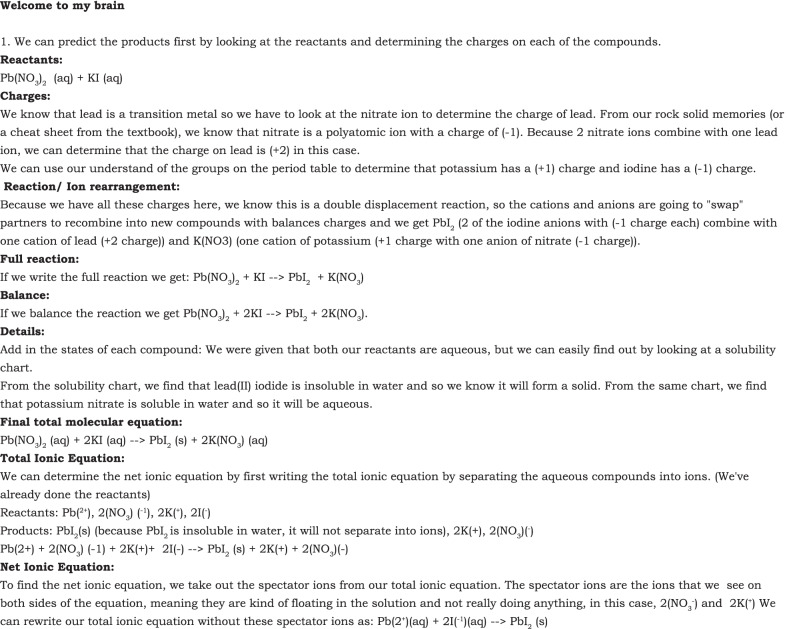
Student response to a problem-solving exercise

These types of exchanges were also noted when students were practicing methods of self-evaluation during problem-solving. Explaining how to predict the products of a precipitation reaction, Tony posts a detailed response entitled “Welcome to my Brain” (Fig. 4). Several students went on to reply to the explanation. Sue writes, “Awesome stuff here. Your brain seems orderly and concise. Your breakdown of the precipitation equation is awesome!” Erin expresses how the detailed explanation was helpful.

I can’t believe how organized you are in your thought process and your problem-solving... It’s helpful to see the way you laid everything out in such detailed steps. I hope with more practice I’ll eventually get to the point of being able to easily approach and separate long problems into multiple small steps.

Billie (replying to Tony): The way you lay out your steps is really easy to follow! I appreciated all of them, but step three was especially nice to take a step back and see how someone tackled it.

Sue, Erin and Billie each refer to how Tony has “broken down” or “laid everything out” as Tony is modeling good problem-solving strategy. There were a dozen instances of students commenting on a peer’s strategy use or approach to problem-solving. A few more representative examples are shown below.

Erin (Week 7; adjusting goals and schedules): Wow Billie this is a great schedule!... I think I’m going to draw up a better schedule for myself for the month of April and use some elements of yours as inspiration **(Basic theme: *I should try that*).**

Sue (Week 8; practicing self-evaluation by “teaching” problems to a peer): I think the way you approach the practice problems to assess your understanding of the larger concepts they apply to is spot on and something I should do more **(Basic theme: *I should try that*).**

Michael (Week 2; Mindsets): I like how you were able to apply that to school. I think you bring up a great point that each course can be viewed as building a skill. That is a mindset that I need to develop **(Basic theme: *I like that*).**

Throughout the module students engaged in conversations which extended beyond the original focus of the modules. They were observed elaborating on and exchanging study strategies and offering and receiving support. Surprisingly, students were open about personal challenges and there were instances of students bringing personal problems to the forum for discussion.

### Organizing theme: bringing problems to the group

This organizing theme describes instances in which students provide one another with encouragement or emotional support. Basic themes included: *sharing a personal obstacle, relating with an obstacle, empathizing, and supporting & encouraging* (Fig. 3).

### Basic theme: sharing a personal obstacle

An example of this type of peer support is observed when Billie shares a negative interaction they had on campus. The full description of the “bad experience” has been edited to maintain the student’s privacy. However, as indicated in the text below, it was quite jarring to the student.

Billie: So far I haven’t done much to prepare... It [place to study] had been the tutoring center from last summer to current, but I had a bad experience a few days ago that I've been trying to shake **{Basic theme: *sharing a personal obstacle*}** I try to return to the same seat and do things in the same order so help create a proper mindset for productivity and the almost-quiet mixed with the ability to ask for help had been doing me well, but lately I’ve been nothing but anxious.

Several students go on to offer statements of support.

Darnell: It's hard to find the time when you've got those packed days. Wednesdays are the worst for me and trying to study on a Friday is impossible **{Basic theme: *relating with an obstacle*}.** My brain is just a strainer at that point. I'm so sorry to hear that someone was harassing you while you’re trying to study **{Basic theme: *empathizing*}.** Would studying with others help alleviate some of this anxiety?”

Tony: I also struggle with trying to budget in daily study time **{Basic theme: *relating with an obstacle*}** I am so sorry you were harassed! **{Basic theme: *empathizing*}.** That is absolutely unacceptable and you should not feel anxious or uneasy, especially in a place you’ve been utilizing!... If you need anything, please please, please reach out **{Basic theme: *supporting & encouraging*** Have you considered using a planner? I thought they were dumb until I started using one and now I can’t function without it **{Basic theme: *independently introduced strategy*}**... Sounds like you’re learning a lot and have a lot of good habits going for you! Good luck!

Here Tony is encouraging Billie to “reach out” if they need anything while also suggesting that they use a planner to stay organized. Darnell suggests that Billie study with a partner. The interactions above are an example of the organizing theme, *Bringing Problems to the Group*.

In the previous example, the problem brought to the group was an external conflict. At other times, internal problems were shared. This is the case as Erin discusses “feeling discouraged” at times.

Erin: I feel encouraged by the handout, because it shows that we are capable of improvement, and capable of things we weren’t, previously. I am definitely guilty of feeling discouraged and not “smart” when I can’t do things immediately and can’t figure out things like how to solve problems or find information immediately**.** It is nice to know that I am not alone in this and in fact it is normal to need to work on things to improve at them. It is also reassuring to read that things get easier as you exercise and strengthen your brain.

### Basic theme: relating with an obstacle

Darnell empathizes with this obstacle writing, “I completely understand not feeling smart when I can’t get something straight away. I'm really hard on myself but have had to learn to be kinder to my mind and remember that learning is lifelong.”

Throughout the module, students empathized with each other and expressed statements of support as shown above. In another post, Darnell shares the following when discussing the role of preconceptions in learning:

Darnell: My preconceived notions of how the world works definitely affect my learning. I took chemistry in high school and was taught oddly and didn’t quite understand the concepts then but somehow cemented wrong facts into my head. Therefore, I’m having to completely wipe away my previous assumptions.

Billie (replying to Darnell): I can relate to what you wrote about having preconceived notions that effect your performance now. It’s difficult to wipe away previous assumptions and to put a proper amount of effort towards something you already somewhat know. I hope the article can prove useful for us both in all of our classes.

Billie empathizes with having to “wipe away previous assumptions” acknowledging that it can be “difficult”. Sue also acknowledges the need to start “fresh” sometimes, offering advice to have an “open mind about any subject” to better evaluate where one stands.

Sue: I couldn’t agree more with your post. Math and science are two subjects that do not come easily for me and require a lot of dedication. I think that having an open mind about any subject is the most important basis to learn more. It helps let us realistically evaluate where we stand on a topic and sometimes that means starting fresh.

### Basic theme: supporting and encouraging

This theme describes how students encouraged one another to stay the course, or at times offered support that extended beyond written encouragement to offers to meet outside of class. During the second week of class, Michael responds to a student who views “each course as building a skill” stating that this is a “mindset that I need to develop”. Colin encourages Michael to maintain this outlook, writing, “I think your mindset is the best one you could have right now…I think if you keep going and not give up, you will do whatever you put mind into. Good luck!”.

During another exercise, Michael expresses that they “learned a lot” and that they “never knew how many strategies there were while reading a textbook”, concluding that they “definitely need” to work on reading strategies.

Billie (replying to Michael): If you ever want someone to talk about the material with, that's actually the strategy that I employ the most! It’s great for helping with memory retention or finding flaws in your thoughts that you didn’t notice you had. It can be a little wild to suddenly see so many strategies when you haven't considered it before. I hope that learning about them helped you!

Here Billie has independently offered to talk over material, though Michael does not identify talking through the text with someone as a strategy they considered adopting. Throughout the module students used the discussion board to bounce ideas off one another (*Cognitive Exchange*) and to share both personal and academic obstacles (*Bringing problems to the group*). In this way students used the forum to create a space which offered both cognitive support and emotional support which is described by the global theme *Support Network*.

### Exit survey

In the current study, a natural question is: To what extent do students continue to use strategies that were introduced? Exit surveys were administered to students to gauge the extent to which they changed their study habits over time. Unfortunately, of the ten students who consented to participate and remained active in the course, only five submitted the exit survey. In one question students were asked: “How has the method that you use to study changed over the semester? Explain.” Three of the five students indicated that the COVID pandemic interrupted their normal study routine. Two students indicated that their habits had changed for the better. Colin writes, “It did change. I increased my hours of studying as well as my techniques.” Sue was more specific writing, “I have. I was self-evaluating without realizing it, but now I practice it intentionally and set aside time for it.” Students were also asked to: “Comment about how speaking in class compared to 'speaking' in the discussion board.” Two students expressed being less anxious about participating online. Sue writes: “I am not comfortable speaking in class most of the time. I tend to be anxious unless I feel the environment is welcoming and I have an idea of what we are talking about. Discussion boards do not raise the same general feeling of being uncomfortable or put on the spot.” Darnell is more emphatic writing, “I absolutely love the discussion board. I feel so much more confident to put out my own thoughts and understandings. It’s nice to see and comment on other’s work and see what they have to say.” For the few students who did provide comments, there is some indication that they are now more aware of metacognitive strategies and committed to applying them. Further, some students express the idea that the discussion board allowed them to interact with peers more comfortably.

## Discussion

Metacognition encompasses more than skills and regulation of those skills. Flavell’s original definition of metacognition includes metacognitive knowledge which includes knowledge of oneself as a learner. This manuscript emphasizes that student beliefs about themselves as learners or their beliefs about effective strategies do impact how they approach tasks. To have influence over students’ evolving beliefs, instructors must recognize students’ self-perceptions and beliefs about learning as part of their prior knowledge. Ideally there would be a mechanism in place to confront beliefs that negatively impact behavior while providing alternative perspectives that will lead students to behaviors that will result in learning. The framework presented here engaged students’ beliefs about learning in such a way that they were willing to consider alternative perspectives. As described previously, there were several occasions on which students described feeling “not smart” or “frustrated”. As Colin says, “my mindset was very clear on the fact that there are two options out there: naturally smart or NOT”. This student described having a fixed mindset about their abilities, however, when this belief was confronted, they ultimately appear to have accepted an alternative and more productive perspective. Also, for students to be more metacognitively aware, they need to be consciously engaged with both learning as a process and the study strategies that they employ. The module presented here was effective at bringing awareness to strategies. This is evident by how frequently students reported a recognition of strategies and a desire to change aspects of how they study.

Some students reported never being exposed to certain strategies and concepts presented while for others, the explicit focus on metacognition reminded them of strategies they had been exposed to previously and using unconsciously. Erin describes the latter explaining, “I think that to be a better student and get more out of my time with the text/material, it would be helpful for me to deliberately commit to using a few strategies and try to build it into more of a habit.” This is an apparent transition from an implicit (tacit) understanding lacking an explicit structure to an informal understanding regarding strategy use. These types of transitions are consistent with how, according to Schraw and Moshman (1995) “[i]nformal metacognitive theories allow learners to reflect purposefully and systematically on their performance and in turn to use this information to modify or redirect their future performance” (p.359).

Regarding the first research question, the results of this study indicate that using a discussion-based metacognition module does help students adapt their metacognitive knowledge. The module presented here was able to inform students of new concepts and strategies while providing a structure for students to “reflect purposefully” about the strategies they are currently using. With respect to the second research question, results indicate that the metacognition instruction model presented here does serve as a catalyst for students to incorporate effective study strategies as long as they are engaged with the module material. The evidence here is weaker regarding whether students will continue in the future to incorporate new strategies learned via the module. However, what is clearer is that given the opportunity, students earnestly reflected on their current strategies and considered the benefits of adjusting these strategies.

Finally, the design of this intervention provides students with space and direction to reflect on their own cognition and engage in conversations with their peers. Pintrich (2002) describes the benefits of this type of “shared language and discourse about cognition”, saying:

As they hear and see how their classmates approach a task, they can compare their own strategies with their classmates’ and make judgments about the relative utility of different strategies. This type of discourse and discussion helps make cognition and learning more explicit and less opaque to students, rather than being something that happens mysteriously or that some students ‘get’ and learn and others struggle and don’t learn. (p.223)

Erin expresses unpacking this mystery when they write, “It is nice to know that I am not alone in this and in fact it is normal to need to work on things to improve at them.” Several examples of students comparing their own strategies with their classmates and making “judgments about the relative utility of different strategies” have been presented. Facilitating a shared discourse around cognition is especially important in the community college setting. On average community college students have more demands on their time as many are balancing work and family commitments. This makes study groups (where presumably some of these exchanges would occur) more challenging to schedule and less spontaneous. The current study describes some benefits of facilitating a discussion centered around cognitive strategies and demonstrates that it need not be cumbersome. This is a significant issue as time constraints and inadequate resources have been identified as barriers to the implementation of evidence-based instructional practices (Shadle et al., 2017). In their investigation into chemistry instructors’ perspectives on metacognitive development, Heidbrink and Weinrich (2021) report that instructors interviewed in the study indicated that they wanted more training on how to develop metacognition in their courses and named time and institutional constraints among barriers to doing so. The framework described here represents a metacognition instruction model that is easy to implement and requires minimal training.

As discussed previously, there are few metacognition instruction models presented in the chemistry education literature. Furthermore, according to a review by Lavi and coworkers (2019), only a narrow range of sub-aspects of metacognition have been addressed in the past two decades, with evaluation and planning being most frequent. An important goal achieved by the work reported in this article then is demonstrating that a more comprehensive metacognitive curriculum can be implemented alongside course content instruction. This metacognitive curriculum incorporates knowledge and regulation of cognition, learning strategies, and importantly, personal beliefs about learning. The format of implementation, parallel presentation with discussion-board interaction, makes the added time and effort manageable for both the instructor and students.

### Limitations

Several considerations and observations will affect the value to STEM instructors of the results reported here. Regarding the number of students that withdrew or stopped attending it should be noted that general chemistry I sections taught by the first author had a DFW rate of 36% (for 2017–2018) which is typical for this course at this institution. Interestingly, none of the students who stopped attending (*N* = 4) ever submitted a discussion post. Of the students who withdrew, most (*N* = 3) submitted two posts and one student submitted three posts. This suggests that early student engagement may be critical if the metacognition module is to have value for supporting student persistence. A second consideration regarding interpreting the data is that because of the small number of participants in this study, it was not practical or useful to look at different impacts based on racial, gender or any other demographic distinction. A subsequent study with more students and with more sites implementing the TALK model would afford an opportunity to explore disaggregated data. Third, further research is needed to determine whether students continue to incorporate strategies that they identified as important in subsequent courses. Lastly, as this study engaged a small number of students at a single institution, one can challenge how well the module might generalize to other courses and institutions. We can report that the TALK module has been disseminated to two other science instructors teaching Introduction to Engineering (same institution) and Organic Chemistry I (different institution; four-year university). The adoption of the module was straightforward with only minor changes needed to replace general chemistry-specific content.

## Conclusions

Based on the results of this study a model has been presented that successfully engages students in building their metacognitive awareness and skills and which is easy for instructors to implement. As mentioned in the introduction, this fulfills a documented need for additional tested metacognition instruction models. A critical consideration here is the ability to encourage students to think about their current practices and beliefs about learning. Instructors who observe students making self-deprecating statements or expressing misconceptions around cognition might consider implementing the framework described here.

This study demonstrates a systematic, multi-faceted approach to introducing students to thinking about their thinking. The module is embedded in the curriculum, delivered through the course management system, and is simple to disseminate and implement.

## Supplementary Information

**Additional file 1: **TALK module overview.

**Additional file 2: **Discussion-post rubric for TALK module.

**Additional file 3: **Exit survey distributed to students.

**Additional file 4: **Codebook.

## Data Availability

The datasets generated and/or analyzed during the current study are not publicly available due to concerns over protecting participants’ privacy, but are available from the corresponding author on reasonable request.
