# Supplementary material for: Beyond “study skills”: a curriculum-embedded framework for metacognitive development in a college chemistry course
Source: Int J STEM Educ. 2022 Sep 24;9(1):61. doi: 10.1186/s40594-022-00376-6 (PMC9510263; doi:10.1186/s40594-022-00376-6)
Supplement: Supplementary file 1 — Additional file 1: TALK module overview. [file 40594_2022_376_MOESM1_ESM.pdf]

Beyond "Study Skills": A Curriculum Embedded Framework for Metacognitive Development in a College Chemistry Course

Sonja Gamby[1,2] , Christopher F. Bauer[1*]

1. **Natural Sciences, North Shore Community College, Danvers, MA 01923, United States**
2. **Department of Chemistry, University of New Hampshire, Durham, NH 03824, United States**

Corresponding Author

***Email: Christopher.Bauer@unh.edu**

Supplementary Information 1

TALK Module – Weekly Resources and Prompts

Talking About Learning is Key (T.A.L.K) Module Overview

Phase I

Week 1: What is your model of success?

What is success to you and what determines if one will be successful? According to Dr. Angela Duckworth it's Grit. Watch the following video and respond to the questions below.

Resource(s): Grit: the power of passion and perseverance, Angela Lee Duckworth accessed at http://www.youtube.com/watch?v=H14bBuluwB8

Prompts:1 . How do you define success and what do you think one's success is determined by? 2. Do you think Duckworth would say that grit was the only thing necessary to succeed? Explain. 3. What are your initial thoughts about what you have seen?

Week 2: What is your model describing your ability to learn?

This short article explains how connections in the human brain grows in response to learning new things. The video below summarizes a book, Growth Mindset, written by Dr. Carol Dweck. It summarizes some of Dweck's findings as she investigated the relationship between one's mindset and their ability to accomplish tasks.

Resource(s): You can grow your intelligence, Mindset Works accessed at https://www.mindsetworks.com/websitemedia/youcangrowyourintelligence.pdf ; Growth Mindset, by Carol Dweck (animated summary) accessed at http://www.youtube.com/watch?v=EyIF5VUOJc0 ; Bloom's Taxonomy (LSU Center for Academic Success) accessed at https://www.youtube.com/watch?v=Qfp3x_qx5IM

Prompts: 1.Were you familiar with the idea of the brain growing in response to learning before reading the handout? 2. Describe any initial impressions you had about the reading. For example, did you feel encouraged or about the same as before? Do you have any questions about the topic?

Week 3: What is your model for learning new information?

Science, in general, is the systematic way in which we come to understand the world around us. Chemistry is the study of substances, their properties and how they are converted into other substances. Biology is the study of living organisms. What does science have to say about learning? There is a Science of Learning which includes a body of research describing how individuals acquire knowledge.

Resource(s): Understanding the Science of Learning, Sonja Gamby, adapted from How People Learn, National Academies Press (PDF attached).

Prompts: There are three main messages from the excerpt in part I. Choose one of the "key points" discussed to reflect upon (in 2-3 sentences). Was this something that has always been obvious to you? Have you noticed any of these strategies being used throughout your education? Describe your impressions about what you read. 2. Below you can find three of the Chapter 2 Learning Objectives from your text. Classify the following Chapter Objectives according to Bloom's Taxonomy: Working with Atomic Numbers, mass Numbers and Isotope Symbols; Predicting the Charge of Ions; Converting Between Mass and Amount (in Moles).

Phase II

Week 4: What is your model for extracting meaning from a text?

Have you read the chapter?? Too often in science classes, textbooks end up as paper weights instead of learning aids. I can't understand the text! That is a common sentiment among students. Gathering meaning from text requires active monitoring and effort.

I. This week, you will complete the Metacognitive Awareness of Reading Strategies Inventory attached below.
II. After you complete the inventory, you will score it using the attached rubric.
III. Lastly, answer the following questions regarding the inventory

Resource(s): Metacognitive Reading Awareness Inventory, Mokhtari, K., & Reichard, C. (2002). Assessing students' metacognitive awareness of reading strategies. Journal of Educational Psychology, 94 (2), 249-259. (PDF attached)

Prompts: 1. Were there any strategies that you were unfamiliar with or strategies that you don't use frequently? A list of strategies is fine. 2. When you calculated your scores for the inventory, which sub-scale was the highest? Lowest? 3. Was there any strategy that you read which you plan to use more frequently? 4. Describe any thoughts or feelings you may have had while or after completing the inventory.

Week 5: What is your model for how you regulate your studying?

Studying is cyclical and requires substantial effort. On the first day of class, your instructor likely explained that you should spend 6-8 hours outside of class studying. You may have been thinking – *That sounds great but I could never find time for that!* Well, you probably DO have time for it...if you use your time efficiently. Self-regulated learning is related to metacognition and encompasses many skills that help one regulate, or control, their own learning. Read the following pdf and answer the questions below.

Resource(s): Metacognition and Self-Regulated Learning, Sonja Gamby (PDF attached)

Prompts: 1. How would you structure your particular schedule to account for the recommended 8 hours of study time. For example, Tuesdays and Thursdays after class from 2-4pm and Saturday and Sunday from 8-10am. 2. Describe any goal setting and planning that you used this week in preparation for your quiz. Do you plan to make any adjustments to your goal setting and planning? 3. Describe the type of environment you prefer to study in? Is your preferred setting an effective place to study? 4. Have you considered evaluating yourself before this course? If so, what techniques did you use? If not, do you have any concerns/questions about your ability to self-evaluate?

Week 6: What is your model for how you study for an exam?

How do you study? Are there any rules? When you sit down to study, do you have a plan? The Center for Academic Success at LSU has created a recommended "Study Cycle" for their students. The short video below explains the cycle and the pdf is included below for your reference.

Resource(s): Study Better in College, LSU Center for Academic Success accessed at https://youtu.be/ppPIYbe3D68

Prompts: Do you currently "preview" before lecture. If so, explain what you do. If not, would you consider setting aside 5-10 minutes to preview before lecture? Why or why not? 2. As it relates to the study cycle, do you currently study in a similar fashion? Do you have specific goals during study sessions? Is there a part of the study cycle that you could emphasize more in your personal studying sessions?

Week 7: Some helpful strategies.

This week we will review three more tools to add to your "Metacognitive Toolbox". Questions follow below.

Resource(s): PDF (attached)

Prompts: 1. What were your general thoughts regarding the note taking system described above? 2. A key component of the Cornell Note Taking System is the "Summary" section. Do you currently summarize your notes? If so, how soon after class do you do so? Do you have an entirely different system for taking notes? Please share! 3. In addition to the conceptual understanding needed to understand chemistry, there is also a good deal of memorization required. What techniques do you use to help you remember?

Phase III

Week 8: Revisiting self-regulation.

For the next few weeks we will be utilizing some of the strategies discussed thus far. This week we will focus on goal setting and planning. *This is weirdly, very timely!*
This has been quite the semester. You likely had a plan that worked for you and as of 2 weeks ago it was blown to pieces. How are you planning on adjusting?
First, what is your goal? Second, what is your detailed plan to accomplish this goal? Previously, you provided a general schedule for studying time.
This week, you will be creating a detailed schedule, meaning, what you will do (i.e review PPT, complete lecture assignment, begin HW, finish HW, perform lab, eat, walk the dog, teach the kid, etc..)

Please upload a copy of your goals for the week along with a detailed schedule (you can type this here or write it by hand and take a picture).

Week 9: Revisiting self-evaluation. How do you know what you know?

During Week Five we reviewed some general metacognitive skills involving self-evaluation. Two of the strategies mentioned were:
walking through a problem, verbally noting the steps required to solve the problem
pretending to teach someone else a concept

This week apply these strategies to the problems below. Either write out a detailed explanation in complete sentences or talk through the explanation and capture it with a recording from your phone/computer. **Respond to all three questions.**

Questions:
1. When aqueous solutions of lead(II)nitrate $Pb(NO_3)_2$ and potassium iodide (KI) are mixed, a solid precipitate forms. Explain (in detail) how to predict the products that form, and write the total molecular and net ionic equations.
2. What is the oxidation number of S in SO_3^{2-} ?
3. Given the following reaction:
$6\,Li(s) + N_2\,(g) \rightarrow 2\,Li_3N\,(s)$

What mass of Li (in grams) reacts completely to form 58.5 mL of N_2 gas at 35 C and 1.1 atm?

Week 10: Revisiting extracting meaning from a text.

This week, you are tasked with reading a selection from your book and paraphrasing the relevant information. For sections 8.5 - 8.8 [from Tro, Chemistry: A Molecular Approach. Chapter 8: The Quantum Mechanical View of the Atom] summarize each section in 3-4 sentences *using your own words*. If you prefer, you can also utilize bullet points. Do not worry about the summaries being 'perfect', the main goal is to extract the relevant information. Also, as you read, make a note of any strategies you are using. Are you writing down unfamiliar words/phrases? Are you making note of any questions you might have?
