# Supplementary material for: Beyond “study skills”: a curriculum-embedded framework for metacognitive development in a college chemistry course
Source: Int J STEM Educ. 2022 Sep 24;9(1):61. doi: 10.1186/s40594-022-00376-6 (PMC9510263; doi:10.1186/s40594-022-00376-6)
Supplement: Supplementary file 2 — Additional file 2: Discussion-post rubric for TALK module. [file 40594_2022_376_MOESM2_ESM.pdf]

Beyond "Study Skills": A Curriculum Embedded Framework for Metacognitive Development in a College Chemistry Course

Sonja Gamby[1,2] , Christopher F. Bauer[1*]

1. **Natural Sciences, North Shore Community College, Danvers, MA 01923, United States**
2. **Department of Chemistry, University of New Hampshire, Durham, NH 03824, United States**

Corresponding Author
***Email: Christopher.Bauer@unh.edu**

Supplementary Information 2
Discussion Post Rubric

Each discussion post is graded out of 10. The original response is worth 7 points, the reply is worth 3 points. General Guidelines for posts: You are not obliged to agree with everything you encounter. In fact, contrary opinions are welcomed. All that is asked is that you reflect thoughtfully and honestly and try and support that position from your experience and material in the reading/video. Replies should address specific points from the original posts. You can agree, disagree or anything in between so long as all replies are courteous and respectful.

	Discussion (Out of 7)	Response (Out of 3)
**Excellent** All questions were addressed in at least 2-3 sentences. At most minor grammatical or spelling errors. Thoughtful, reflective response with specific examples from the content.	7	3 – Reply specifically addresses points brought up in the original post.
**Very Good** All questions answered. Overall, a thoughtful response that may contain several grammatical and spelling errors.	6	
**Good** All questions are answered, though in a superficial manner. Response has some relation to the content. May contain spelling or grammatical errors.	5	2 – Reply related to the content of the original post.
**Poor** Questions are answered with one- or two-word responses such as “yes” or “I agree” with little to no relation to the content. Grammatical errors are so severe that the post is incomprehensible.	1-4	
**Not submitted**	0	0 – No reply posted; Reply is not respectful.
