# Supplementary material for: Beyond “study skills”: a curriculum-embedded framework for metacognitive development in a college chemistry course
Source: Int J STEM Educ. 2022 Sep 24;9(1):61. doi: 10.1186/s40594-022-00376-6 (PMC9510263; doi:10.1186/s40594-022-00376-6)
Supplement: Supplementary file 3 — Additional file 3: Exit survey distributed to students. [file 40594_2022_376_MOESM3_ESM.pdf]

Beyond “Study Skills”: A Curriculum Embedded Framework for Metacognitive Development in a College Chemistry Course

Sonja Gamby[1,2], Christopher F. Bauer[2*]

1. Natural Sciences, North Shore Community College, Danvers, MA, 01923, USA
2. Department of Chemistry, University of New Hampshire, Durham, NH, 03824, USA

*Corresponding Author email: Christopher.Bauer@unh.edu

Supplementary Information 3

Exit Survey

1.Earlier this semester you were asked about what factors contributed to success. Have your opinions changed in this regard?

2. A word that popped up a lot this semester was "frustrating". How has your opinion regarding the subjects you struggle with changed over the course of the semester?

3. Can you see yourself mastering chemistry or another subject that you may have struggled with at the start of the semester. (Feel free to declare mastery already!)

4.Has the method that you use to study changed over the semester?

5. Think of a time you may have been stumped early on in the semester, has the way you approached problems changed at all? What do you do when you are "lost"?

6. During the semester we spoke about self-evaluating. Have you incorporated any self-evaluation into your studying? Please explain.

7. Comment about how speaking in class compared to 'speaking' in the discussion board

8. Talk about what it meant to “go to class” before spring break and after. In other words, describe for THIS class, what you would do for “a class” before spring break. Then describe what you do now when you “go to class”.

9. How, if at all, has your approach to studying changed in this course since it moved online?

10. To what extent would you have described yourself as being a member of a community of learners in the class before spring break, and contrast that with your feeling now that things are on-line. Were you more "known" at one time or the other? Did you feel isolated or anonymous at one time or the other? Please be specific about what led you to these perceptions.
