# Supplementary material for: Beyond “study skills”: a curriculum-embedded framework for metacognitive development in a college chemistry course
Source: Int J STEM Educ. 2022 Sep 24;9(1):61. doi: 10.1186/s40594-022-00376-6 (PMC9510263; doi:10.1186/s40594-022-00376-6)
Supplement: Supplementary file 4 — Additional file 4: Codebook. [file 40594_2022_376_MOESM4_ESM.pdf]

Beyond "Study Skills": A Curriculum Embedded Framework for Metacognitive Development in a College Chemistry Course

Sonja Gamby[1,2] , Christopher F. Bauer[1*]

1.Natural Sciences, North Shore Community College, Danvers, MA 01923, United States
2.Department of Chemistry, University of New Hampshire, Durham, NH 03824, United States

Corresponding Author
***Email: Christopher.Bauer@unh.edu**

Supplementary Information 4
TALK - Codebook

Name	Description
**Metacognitive Awareness (MA-A)**	**This general code relates to items in which students indicate their awareness (or lack of) of the various concepts and strategies introduced.**
1. MA A View of Success	Items here describe statements in which students discussed their view of success.
MA A1	Describes statements in which students indicate that they view success as a process.
MA A2	Describes statements in which students indicate that they view success as an outcome
MA A3	Describes statements in which students indicate that they now have a new Perspectives on Success
2. MA Brain growth and Mindsets (MA-B)	Students indicate their level of awareness concerning how the brain grows and about various mindsets
MA B-	Items here describe statements that students made which indicate that they were unaware of how the brain grows in response to stimuli
MA B+	Items here describe statements in which students indicate that they have an awareness of how the brain grows in response to learning and/or mindsets
MA B++	Items here describe statements in which students indicate that they previously had a vague awareness of mindsets/brain growth and that they now know more about the subject

Name	Description
3. MA C Cognition	Preconceptions, metacognition and the importance of deep conceptual understanding were discussed
MA C1 - deep factual understanding	Items here describe statements in which students discuss the importance of dep factual understanding. Students indicated that this aspect of learning resonated with them.
MA C2 - Metacognition	Items here describe statements in which students discuss metacognition. Students indicated that this aspect of learning resonated with them.
MA C3 - Preconceptions	Items here describe statements in which students discuss preconceptions. Students indicated that this aspect of learning resonated with them.
4. MA D Reading Strategies	Items here describe statements in which students indicated that they are aware of strategies and use them
Awareness MA D -	Students are unaware of most reading strategies presented.
Awareness MA D +	Items here describe statements in which students indicate that they are aware of most/all of the reading strategies but may or may not use them.
5. MA E Self-Evaluation	Students respond to whether or not they self-assess ("self-evaluate"
Evaluation MA E-	Describes statements in which students indicate that they are not currently self-evaluating when they study
Evaluation MA E+	Describes statements in which students indicate that they are currently self-evaluating when they study
6. MA F Previewing and Reviewing	Students discuss previewing and reviewing.

Name	Description
Aware MA F +	Describes statements in which students indicate that they are aware of previewing and reviewing as a strategy
Unaware MA F -	Describes statements in which students indicate that they are not aware of previewing and reviewing as a strategy
7. MA G Explicit Awareness	Items here describe statements that students make expressing the benefit of being explicitly aware of a strategy or concept
8. MA-H New Thought	Items here describe statements in which students express wanting to try a strategy that they had previously not been aware of
9. MA I New Awareness (General)	This describes items in which students express being made aware of a concept or skill for the first time.
10. MA J Elaboration and Translation	Describes statements in which students discuss putting things in their own words as a strategy
11. MA K Revisiting	Describes statements in which students discuss an idea/concept that was previously discussed.
12. MA L Deconstruction	Describes statements in which students discuss breaking material into chunks as a strategy to process it.
13. MA M Organizing and abstracting	Describes statements in which students discuss highlighting and extracting key ideas.

Name	Description
**Metacognitive Experiences (ME)**	**This describes items in which students express any feelings associated with the strategies or concepts introduced.**
14. ME A Encouraged	This describes items in which students discuss if they are "encouraged" or "hopeful" in response to a concept that was introduced.
Yes ME A+	This describes items in which students express being "encouraged" or "hopeful" in response to a concept that was introduced.
No ME A -	This describes items in which students express not being "encouraged" or "hopeful" in response to a concept that was introduced.
16. ME C Motivation	This describes items in which students discuss motivation.
17. ME D Negative Self-Thoughts	This describes items in which students make comments that diminish their intelligence or abilities, or, express being frustrated with their performance.
18. ME E Questioning grit	Students respond to whether grit is the only requirement for success
ME E-	Describes statements in which students indicate that grit is not the only thing necessary
ME E+	Describes statements in which students indicate that grit is the only thing necessary
19. ME F Questioning limits to brain growth	Describes statements in which students independently questioned the limits to brain growth

Name	Description
20. ME G Appraising	Describes statements in which students are apparently weighing the potential benefits of adopting a strategy or concept. This includes statements in which students evaluated the benefits of using a strategy, discussed the intentional use of strategies, or recalled a past academic challenge while discussing a strategy.
21. ME H Personalization	Describes statements which indicate that student connects with material in a personal way as demonstrated by recalling a previous experience or discussing how the concept/strategy is relevant to or could be integrated into their life.

23. ME K Makes Sense	Describes statements in which students indicate that a strategy/concept seems reasonable
**Peer to Peer Interactions (P2P)**	**Items in this category contain instances in which students are interacting directly with one another to empathize, agree or exchange ideas with one another.**
24. P2P-A Agree	This describes items in which students express general agreement with one another in response to a discussion post.
25. P2P-B Empathizing	Describes statements in which students express sympathy/empathy to another student in response to their post.
26. P2P-C I can relate	Describes statements in which students express relating to a challenge presented by a peer.
27. P2P-D I like that	Describes statements in which students indicate that they like a strategy described by a peer.
28. P2P-E I never thought of that	Describes statements in which students indicate that they never considered a strategy/opinion expressed by a peer.

Name	Description
29. P2P-F I should try that	This describes items in which students respond to a strategy posed by a classmate and express a desire to incorporate the strategy/mindset.
30. P2P-G Independently introduced strategy	This describes items in which students introduce a strategy that has not been discussed in the module.
31. P2P-H Supporting & Encouraging	This describes items in which students offer support or encouragement to one another in response to their post.
32. P2P-I Sharing a personal obstacle	Describes statements in which students share a personal concern or obstacle.
